# The Iron–Mitochondria–Oligodendrocyte Axis: An Emerging Metabolic Framework for Alzheimer’s Disease

**DOI:** 10.3390/brainsci16101097

**Published:** 2026-10-12

**Authors:** Xinhao Li, Wanxuan Xiao, Mobai Hu, Li Li, Li Lin

**Affiliations:** 1 Hubei Shizhen Laboratory, Department of Physiology, College of Basic Medical Sciences, Hubei University of Chinese Medicine, No. 16 of Huangjiahu West Road, HongShan District, Wuhan 430065, China; 2022302013195@stmail.hbucm.edu.cn (X.L.); 2022302013116@stmail.hbucm.edu.cn (W.X.); 2022302013129@stmail.hbucm.edu.cn (M.H.); 2985@hbucm.edu.cn (L.L.); 2 College of Acupuncture-Moxibustion and Orthopedics, Hubei University of Chinese Medicine, Wuhan 430061, China

**Keywords:** Alzheimer’s disease, ferroptosis, oligodendrocyte, iron–mitochondria–oligodendrocyte axis, multi-targeted therapy, precision medicine

## Abstract

**Highlights:**

**What are the main findings?**

- This study proposes the “Iron-Mitochondria-Oligodendrocyte Axis”, demonstrating intracellular iron overload triggers oligodendrocyte-specific mitochondrial dysfunction, energy depletion and ferroptosis.
- Iron-provoked oligodendrocyte rupture releases damaged mtDNA and oxidized lipids, activating the microglial cGAS-STING pathway to amplify neurotoxicity and axonal metabolic impairments.

**What are the implications of the main findings?**

- The IMOA framework bridges protein accumulation and neuronal death, shifting AD research focus from protein aggregation to general metabolic dysregulation.
- It supports multimodal diagnostics for ferroptosis-positive AD patients and facilitates multi-target therapies combining iron chelation, mitochondrial protection and remyelination.

**Abstract:**

With the accelerating process of global aging, Alzheimer’s disease (AD) has become an enormous public health burden worldwide. However, its pathogenesis is still not fully understood beyond the classic amyloid-β (Aβ) and tau hypotheses. This review aims to fill this critical knowledge gap by proposing the “Iron-Mitochondria-Oligodendrocyte Axis (IMOA)” as a novel metabolic paradigm for deciphering the complex biological mechanisms underlying AD. Specifically, we explicitly aim to address whether this axis functions as a causal, permissive, or modulatory pathway during the early and progressive stages of the disease. We systematically summarize the reciprocal regulatory interactions within this axis, with a focus on how brain iron dyshomeostasis preferentially impacts mitochondria in oligodendrocytes, which are characterized by high metabolic demand. Notably, the IMOA framework highlights disordered iron metabolism as a potential modulatory factor of neurodegeneration by linking iron imbalance to impaired energy metabolism. Excessive intracellular iron induces mitochondrial dysfunction, insufficient energy supply, and reactive oxygen species (ROS) overproduction, thereby triggering oligodendrocyte ferroptosis and myelin degeneration. These events further aggravate neuronal energy depletion, forming a potentially self-sustaining pathological cascade that may operate synergistically with or upstream of Aβ and tau pathologies. This framework not only bridges the logical disconnect between protein accumulation and neuronal degeneration in the Aβ and tau hypothesis but also provides promising targets for multi-target therapeutic strategies. The IMOA offers valuable insights for improving the temporal and spatial precision of AD diagnosis and intervention. Collectively, targeting the IMOA may represent a transformative strategy for developing precision diagnostics and combination therapies to halt or delay the progression of AD.

## 1. Background

Patients with Alzheimer’s disease (AD) suffer from progressively worsening dementia as the core clinical manifestation, accompanied by impaired quality of life and substantial family caregiver burden, all of which urgently need to be addressed. Accordingly, precise diagnosis and treatment of AD are becoming increasingly critical.

Pathophysiologically, AD was traditionally defined as a continuum of neurodegenerative processes driven by the accumulation of amyloid-β (Aβ) plaques and tau neurofibrillary tangles (NFTs), independent of clinical symptoms. In clinical practice, diagnostic evaluation for AD commonly employs amyloid positron emission computed tomography (PET), cerebrospinal fluid (CSF) Aβ42/40, CSF phosphorylated tau (p-tau)181/Aβ42, accurate plasma biomarkers, or combinations of these modalities [1]. AD is characterized by a biological continuum from an initial asymptomatic stage to advanced neurodegeneration, which may or may not synchronize with clinical impairment [2].

Iron is an indispensable cofactor for fundamental metabolic processes in the brain, driving mitochondrial bioenergetics, DNA repair, and neurotransmitter synthesis. Within the central nervous system, oligodendrocytes emerge as the predominant iron-utilizing cells. They critically rely on mitochondrial iron–sulfur clusters and ATP flux to fuel the massive lipid biosynthesis required for myelin production and axonal metabolic coupling. However, despite their high metabolic demand, oligodendrocyte mitochondria possess relatively weak antioxidant defenses, rendering them uniquely vulnerable to iron-driven oxidative injury and ferroptosis.

Compelling evidence has demonstrated marked iron accumulation in the cortex and hippocampus of AD brains, often colocalizing with Aβ plaques and neurofibrillary tangles [3]. Moreover, mitochondrial dysfunction occurs early in the disease course, frequently preceding Aβ plaque deposition [4]. Notably, myelin dysfunction is now recognized as an upstream driver of amyloid deposition, rather than merely a downstream consequence [5]. Furthermore, recent studies indicate that compromised myelin integrity further exposes axons to iron-mediated oxidative stress, accelerating the collapse of the neuronal network [6].

Together, these findings indicate that our understanding of AD can no longer be restricted to the Aβ and Tau hypotheses. In this review, we propose the iron–mitochondria–oligodendrocyte axis (IMOA) as a new conceptual framework. We focused on oligodendrocytes (OLs), the cells with the highest iron content in the brain, which are responsible for myelination, and aim to explore the relationship among disrupted brain iron homeostasis (at the organoid level), mitochondrial dysfunction, oligodendrocyte injury, and the pathogenesis of AD. Unlike existing broad glial-centric or general neuroinflammatory hypotheses, the IMOA framework specifically articulates how region-specific iron accumulation preferentially injures OLs due to their unique metabolic architecture. This constitutes the unique contribution of our synthesis: it links regional iron overload, OL mitochondrial dysfunction, and ferroptosis as an upstream metabolic cascade. We critically appraise the current literature to clearly delineate established evidence from hypothesized causality, offering a balanced perspective on how this axis contributes to the neurodegenerative cascade.

## 2. Physiological Basis of the Iron–Mitochondria–Oligodendrocyte Axis

**Figure 1:**
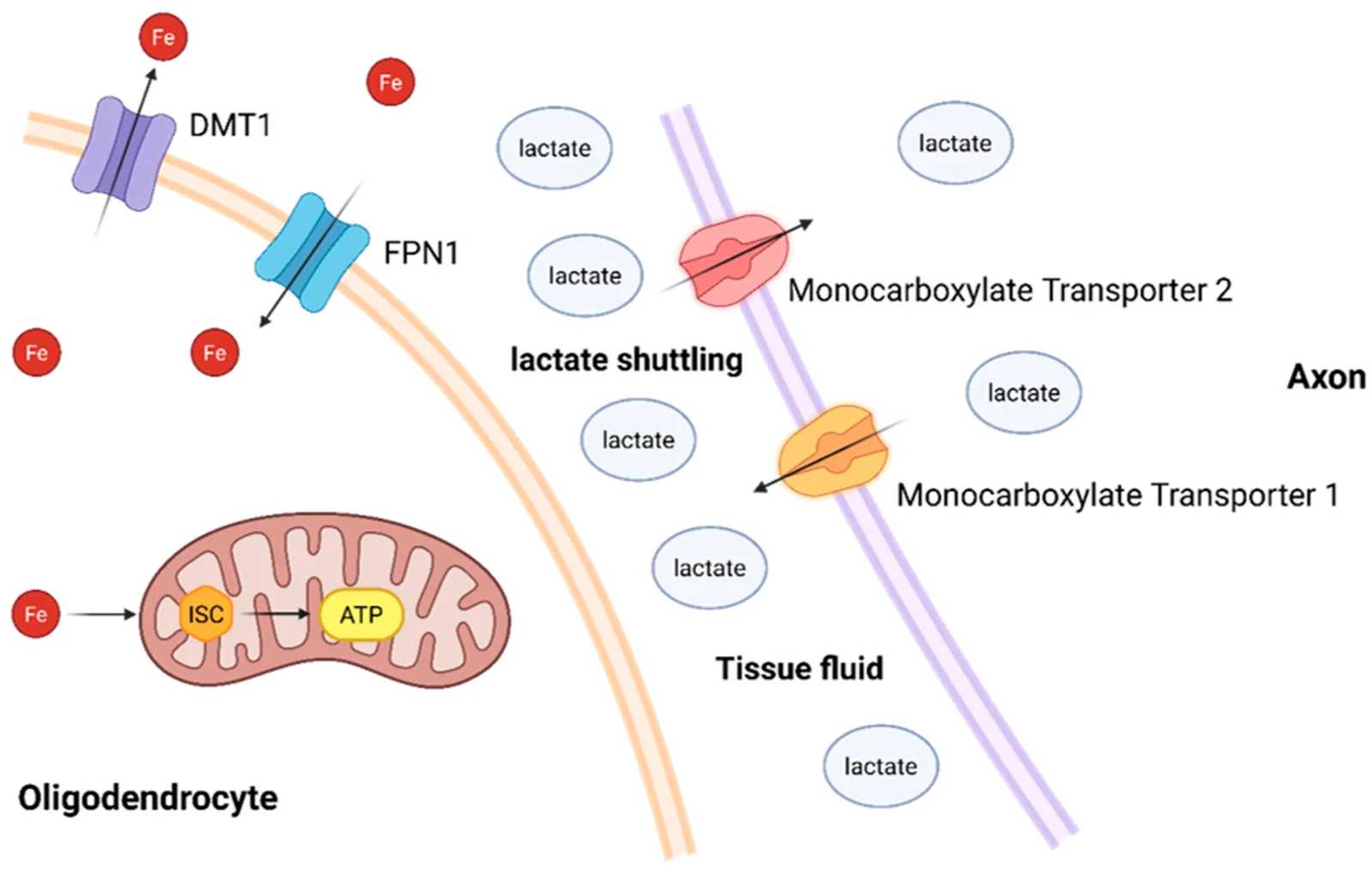
The metabolic triad—physiological basis of the iron–mitochondria–oligodendrocyte axis. Schematic illustration of the metabolic triad in oligodendrocytes. Tightly regulated iron influx fuels mitochondrial bioenergetics, enabling iron–sulfur cluster biogenesis and ATP production. These processes may be indispensable for myelin lipid synthesis and axonal metabolic coupling via lactate shuttling. This functional interdependence may preserve neural circuit stability and saltatory conduction, although the inherently weak antioxidant capacity of oligodendrocyte mitochondria may render the axis vulnerable to metabolic insults. (Note: Core iron trafficking and mitochondrial bioenergetic events are supported by experimental data from oligodendrocyte models; the functional linkage between lactate shuttling and saltatory conduction in this context remains partially speculative.) © 2026 [Xinhao Li, Wanxuan Xiao, Mobai Hu, Li Li, Li Lin]. All rights reserved. Created with BioRender.com.

In the central nervous system, OLs uniquely depend on a tightly regulated iron influx to sustain mitochondrial bioenergetics and iron–sulfur cluster (ISC) biogenesis, as illustrated in Figure 1, such as that orchestrated by the iron responsive element (IRE)/iron regulatory protein (IRP) system and transporters—divalent metal transporter 1 (DMT1) and ferroportin (FPN), processes indispensable for myelin lipid synthesis and axonal metabolic coupling via lactate shuttling [4,7,8,9,10,11,12,13,14,15]. In this metabolic coupling, OLs actively provide essential support to axons by supplying energy-rich metabolites, primarily lactate and pyruvate. While the production of lactate via aerobic glycolysis in OLs is largely iron-independent [16], the massive ATP demands of lipid biosynthesis for myelination strictly require iron-dependent mitochondrial respiration. This intricate functional interdependence constitutes the IMOA, a fundamental metabolic triad essential for preserving neural circuit stability and saltatory conduction [5,6,17,18,19]. However, the inherently weak antioxidant capacity of OL mitochondria renders this axis highly susceptible to metabolic insults [3,20,21,22,23]. Disruption of the IMOA triggers a catastrophic cascade wherein iron dyshomeostasis fuels Fenton-mediated oxidative stress, precipitating severe mitochondrial energetic failure and subsequent ferroptotic cell death [24,25,26,27,28,29,30,31]. Consequently, the collapse of the IMOA has emerged as a prominent modulatory pathway in neurodegenerative disorders, notably AD and Parkinson’s disease (PD) where glial metabolic dysfunction and progressive demyelination exacerbate, and potentially precede, neuronal degeneration [32,33,34,35,36,37,38]. Furthermore, astrocytes play a crucial collaborative role in this network by buffering extracellular iron and mediating the broader astrocyte–neuron lactate shuttle; however, when astrocytic spatial buffering capacity is overwhelmed, OLs are left uniquely exposed to iron-mediated toxicity.

## 3. Pathological Alterations of the Iron–Mitochondria–Oligodendrocyte Axis in AD

### 3.1. Brain Iron Dyshomeostasis in AD

Accumulating evidence indicates that iron dyshomeostasis in AD is characterized by a pathological, region-specific accumulation, predominantly within the hippocampus and cerebral cortex, which strongly correlates with cognitive decline and disease severity [39,40,41].

**Figure 2:**
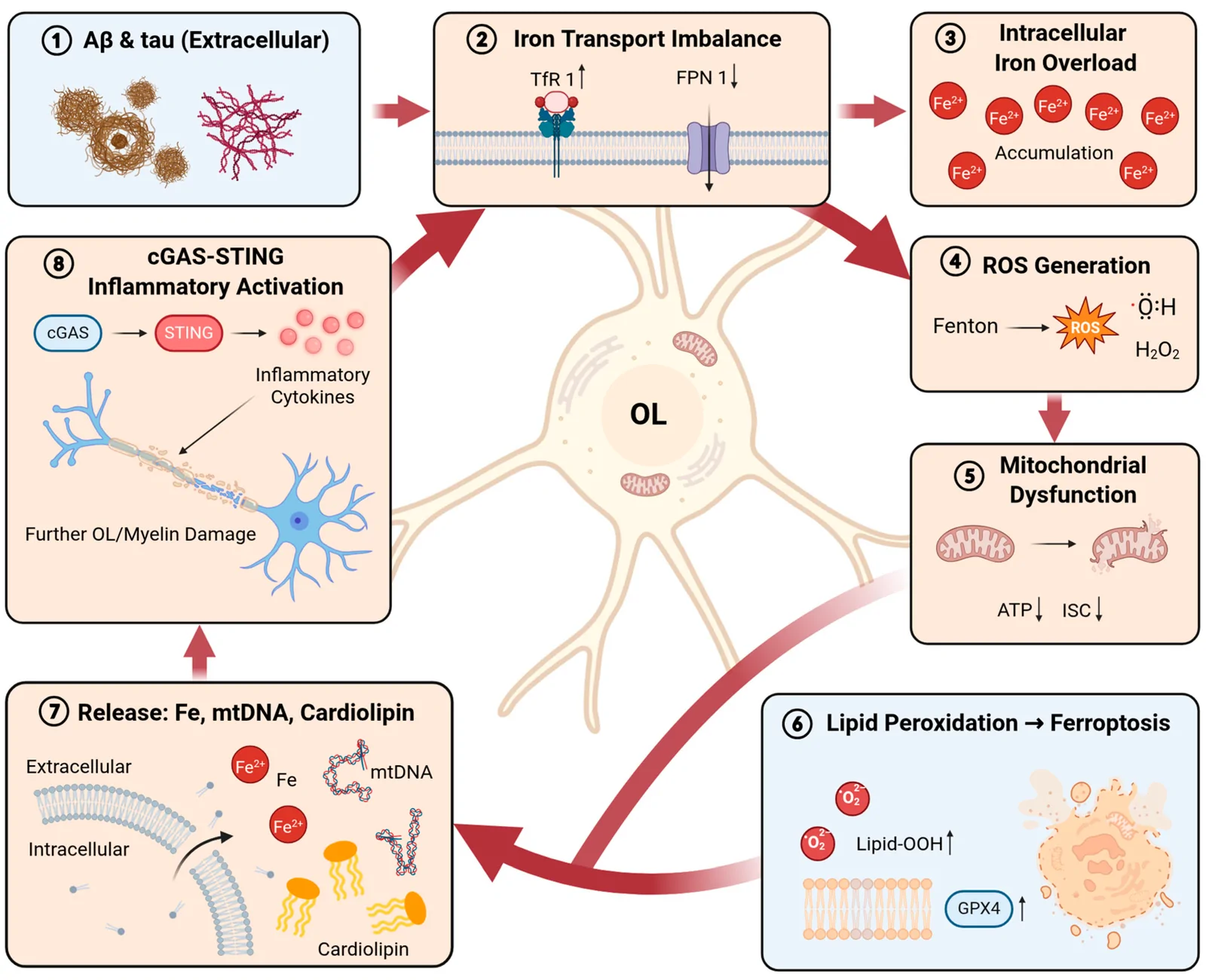
A Self-reinforcing vicious cycle based on iron–mitochondria–oligodendrocyte axis. Oligodendrocyte (OL) iron overload may fuel the Fenton reaction to generate excessive reactive oxygen species (ROS), overwhelming antioxidant defenses such as glutathione peroxidase 4. This may trigger ferroptosis via lipid peroxidation of mitochondrial and plasma membranes, while also suppressing myelin genes such as myelin basic protein, myelin oligodendrocyte glycoprotein and impairing iron-sulfur cluster synthesis. Mitochondrial ATP depletion may further exacerbate ROS production and create a vicious cycle—iron regulatory protein 1-mediated upregulation of transferrin receptor 1 and downregulation of ferroportin 1. Myelin thinning/degradation and lactate shuttle dysfunction may compromise axonal metabolic support, leading to axonal degeneration and neuronal network failure. Damaged OLs release iron, mitochondrial DNA, and cardiolipin, which may act as damage-associated molecular patterns to activate the cyclic guanosine monophosphate–adenosine monophosphate synthase–stimulator of interferon genes pathway in microglia, potentially driving cytokine release. This neuroinflammation may recruit more iron and amplifies OL ferroptosis, forming a self-perpetuating loop. (Note: Selected steps in this schematic are supported by in vitro oligodendrocyte and rodent neurodegeneration data, whereas several downstream cascades remain hypothetical and require further validation in human samples.) © 2026 [Xinhao Li, Wanxuan Xiao, Mobai Hu, Li Li, Li Lin]. All rights reserved. Created with BioRender.com.

As shown in Figure 2, at the cellular level, OLs—the brain cells with the highest baseline iron content—display marked vulnerability to this imbalance, sequestering toxic labile iron within both cytosolic and nuclear compartments [3,42]. This intracellular iron overload frequently colocalizes with Aβ plaques and neurofibrillary tangles, suggesting a synergistic pathogenic microenvironment [33,43]. Mechanistically, pathological iron retention in AD is governed by the systemic dysregulation of cellular iron flux and post-transcriptional control. Aβ oligomers upregulate surface transferrin receptor 1 (TfR1) expression to accelerate iron uptake, while simultaneously destabilizing FPN1, the sole identified cellular iron exporter [11,44,45]. Concurrently, Tau pathology disrupts the axonal transport of amyloid precursor protein (APP), which normally stabilizes FPN1 at the plasma membrane, further impeding iron efflux [46,47]. At the post-transcriptional level, chronic oxidative stress and inflammatory signaling alter the IRE/IRP system. Pathological IRP activation represses ferritin translation while preserving TfR1 expression, culminating in a state of “functional iron deficiency”—wherein iron is trapped in redox-active, labile forms rather than being safely sequestered within ferritin complexes [10,26,32,48].

Despite these compelling associations, it is crucial to acknowledge that much of the evidence linking specific cellular iron overload to pathology stems from in vitro models and transgenic rodents. These models may not fully recapitulate the chronic temporal progression or cellular heterogeneity of human AD. Furthermore, the relative pathogenic contribution of oligodendrocyte-specific iron overload compared to microglial or neuronal iron accumulation remains an active area of investigation that requires validation in post-mortem human tissues. This metabolic failure preferentially compromises highly myelinated projection neurons in these specific regions during the early and prodromal phases of the disease.

### 3.2. Mitochondrial Dysfunction in Oligodendrocytes in Alzheimer’s Disease

Owing to the massive bioenergetic requirements of myelin membrane synthesis, OLs are exquisitely sensitive to mitochondrial perturbations. In AD, OL mitochondria exhibit severe structural abnormalities, including cristae disorganization, swelling, and membrane permeability transition [4,20,49]. At the biochemical level, this is characterized by selective complex IV (cytochrome c oxidase) deficiency, impaired oxidative phosphorylation, and severe intracellular ATP depletion [50,51].

This bioenergetic failure initiates a bidirectional toxic feed-forward loop with iron dyshomeostasis. Mitochondrial respiration defects disrupt ISC biogenesis—a process dependent on functional mitochondrial energetics [10,52]. Defective ISC assembly impairs the activity of key metabolic enzymes and inappropriately activates IRP1 [11,53]. Activated IRP1 subsequently fuels intracellular iron accumulation by upregulating TfR1 and repressing FPN1 [21,54]. In turn, excessive cytosolic iron catalyzes the production of mitochondrial reactive oxygen species (ROS), causing membrane lipid peroxidation and further damaging mitochondrial structural integrity [24,26,55]. This metabolic collapse suppresses lipid and cholesterol synthesis genes via pathways such as the Pyk2-GSK3β-SREBP2 axis [56], directly compromising the cell’s capacity to maintain myelin structural integrity [8,57].

### 3.3. Oligodendrocyte Injury and the Pathological Cascade in AD

Single-nucleus RNA sequencing (snRNA-seq) and spatial transcriptomics have delineated a distinct “disease-associated oligodendrocyte” (DAO) transcriptomic profile in AD brains, characterized by the downregulation of genes essential for myelination, lipid transport, and axonal trophic support [58,59,60,61]. A central feature of OL lineage pathology in AD is the arrest of oligodendrocyte precursor cell (OPC) differentiation. Upregulation of negative differentiation regulators, such as leucine-rich repeat and Ig domain-containing 1 (LINGO-1), prevents OPCs from maturing into functional myelinating cells, thereby impairing endogenous remyelination capacity [62,63,64]. Concurrently, mature OLs undergo accelerated degeneration and apoptosis driven by chronic oxidative stress and excitotoxicity, leading to the rapid loss of major structural myelin proteins including myelin basic protein (MBP), proteolipid protein (PLP), and myelin oligodendrocyte glycoprotein (MOG) [65,66,67,68]. Crucially, quantitative MRI and neuropathological studies demonstrate that myelin breakdown and white matter loss occur during the early, prodromal stages of AD, often preceding overt neuronal perikaryal loss [69,70,71].

## 4. Evidence, Controversies and Unresolved Questions Regarding the Iron–Mitochondria–Oligodendrocyte Axis in AD

**Figure 3:**
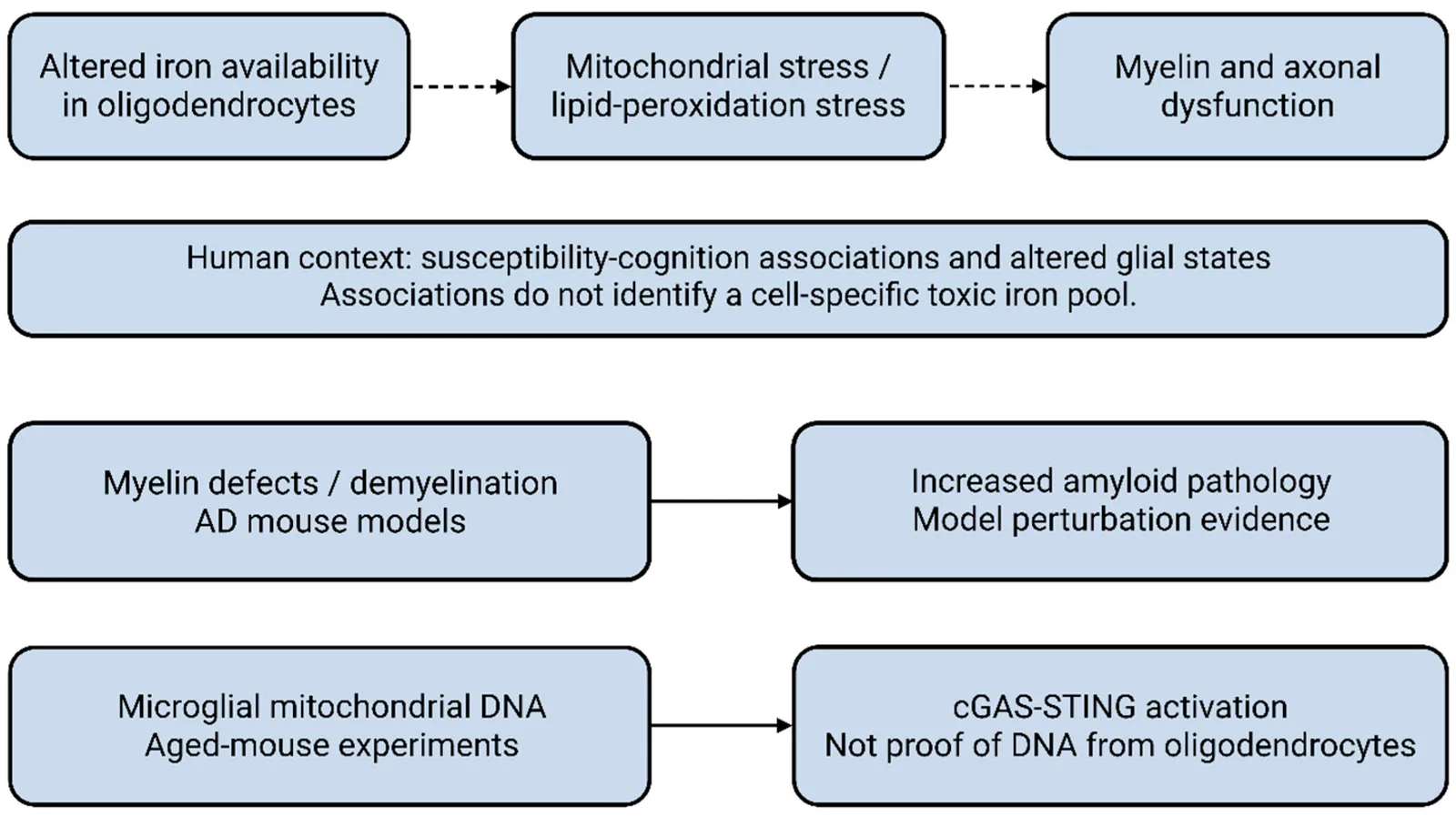
Pathological links and evidence boundaries. Evidence-constrained pathological iron–mitochondria–oligodendrocyte axis model. Dashed arrows represent proposed AD connections that require direct validation. Solid arrows denote independently validated experimental findings: myelin disruption exacerbating amyloid pathology in AD mouse models, and mitochondrial DNA-triggered cyclic guanosine monophosphate–adenosine monophosphate synthase–stimulator of interferon genes (cGAS–STING) activation in aged microglia. Human iron, glial, and clinical associations provide contextual support but do not prove the dashed causal chain. In particular, the transfer of oligodendrocyte-derived DNA to microglia and a complete feed-forward iron–ferroptosis loop remain unproven in human AD. Compensatory metabolism and compartmental iron deficiency represent alternative plausible outcomes and should not be dismissed. © 2026 [Xinhao Li, Wanxuan Xiao, Mobai Hu, Li Li, Li Lin]. All rights reserved. Created with BioRender.com.

As shown in Figure 3, The IMOA provides a refined framework for AD pathogenesis, proposing that ferroptosis may not merely be an incidental epiphenomenon, but rather an active upstream driver of canonical AD neuropathological hallmarks, including Aβ deposition, hyperphosphorylated Tau accumulation, and neurovascular network disruption.

### 4.1. Deep Interconnection Between Oligodendrocyte Ferroptosis and Alzheimer’s Disease Pathological Cascade

#### 4.1.1. Myelin Breakdown, Axonal Starvation, and Axonal Transport Collapse

**Figure 4:**
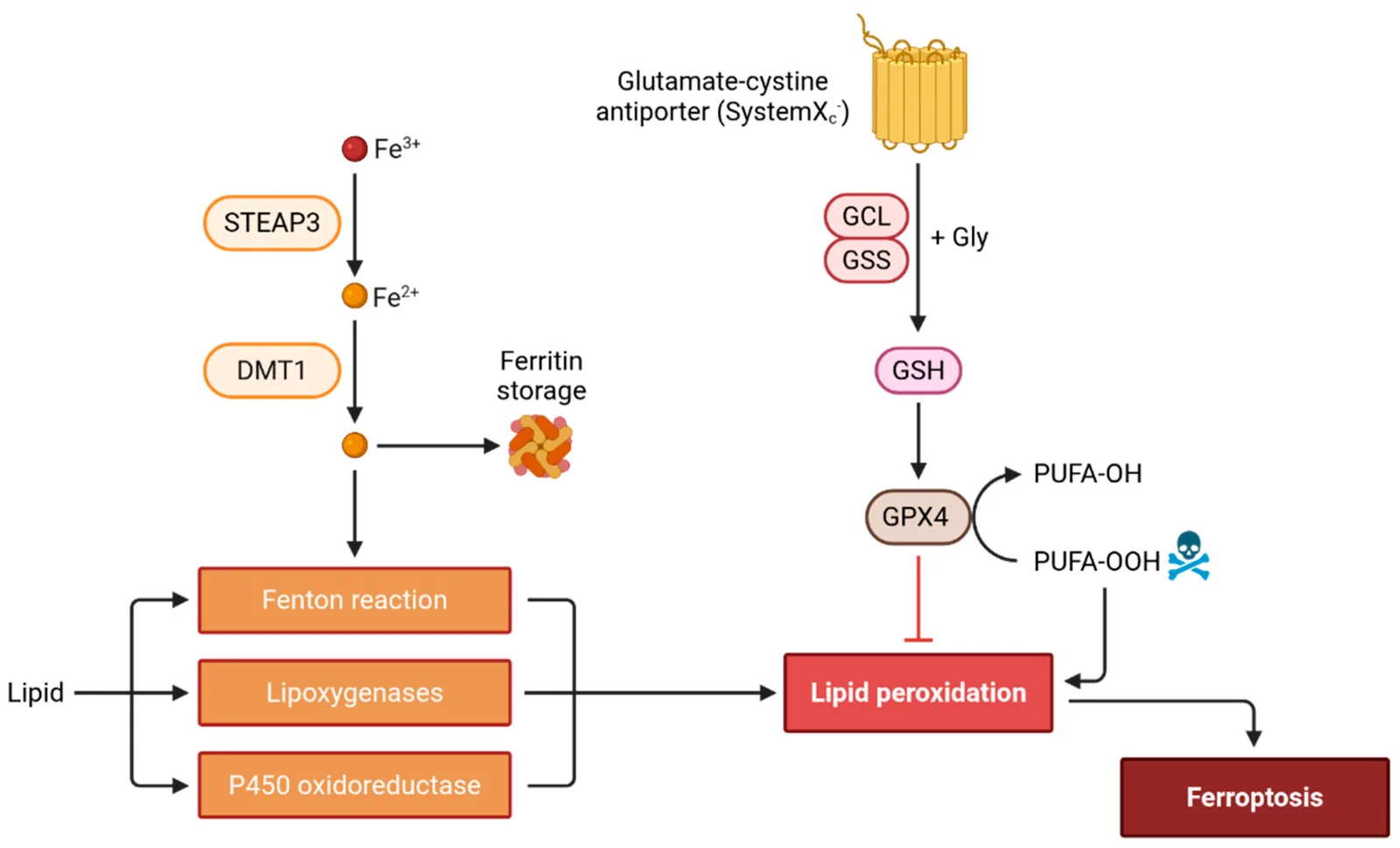
Schematic overview of the molecular mechanisms and regulatory pathways of ferroptosis. Iron metabolism and lipid peroxidation drivers: extracellular Fe^3+^ is reduced to Fe^2+^ by Six-Transmembrane Epithelial Antigen of Prostate 3 (STEAP3) and imported via divalent metal transporter 1 (DMT1) into the labile iron pool (or stored in ferritin). Intracellular Fe^2+^, together with lipids, promotes lipid peroxidation via the Fenton reaction, lipoxygenases, and nicotinamide adenine dinucleotide phosphate-cytochrome P450 oxidoreductase. The cystine/glutamate antiporter (System Xc^−^)/glutathione (GSH)/glutathione peroxidase 4 (GPX4) antioxidant axis: the glutamate-cystine antiporter (System Xc^−^) mediates cystine uptake; cystine is subsequently converted into glutathione by glutamate cysteine ligase and glutathione synthetase in the presence of glycine. GPX4 consumes GSH to detoxify toxic lipid hydroperoxides into non-toxic lipid alcohols, thereby suppressing lipid peroxidation. (Note: Core ferroptosis machinery shown here is well-established across multiple cell types; its cell-type-specific operation in oligodendrocytes under AD pathology remains partially speculative.) © 2026 [Xinhao Li, Wanxuan Xiao, Mobai Hu, Li Li, Li Lin]. All rights reserved. Created with BioRender.com.

As illustrated in Figure 4, OLs possess an inherent biophysical vulnerability to ferroptosis due to their enrichment in polyunsaturated fatty acids (PUFAs), such as arachidonic acid, which are required for myelin membrane architecture. Pathological labile iron accumulation catalyzes the non-enzymatic Fenton reaction, generating highly toxic hydroxyl radicals. Simultaneously, acyl-CoA synthetase long-chain family member 4 (ACSL4) and lysophosphatidylcholine acyltransferase 3 (LPCAT3) incorporate free PUFAs into membrane phospholipids, providing substrates for enzymatic lipid peroxidation [72,73,74]. In the AD microenvironment, this oxidative stress is exacerbated by the inhibition of the canonical cystine/glutamate antiporter (System Xc−)–glutathione (GSH)–glutathione peroxidase 4 (GPX4) antioxidant axis, resulting in uncontrolled membrane lipid hydroperoxides accumulation and ferroptotic cell death [75]. The loss of mature OLs to ferroptosis triggers severe axonal metabolic starvation (as shown in Figure 2). Under physiological conditions, OLs supply lactate and pyruvate to axons via monocarboxylate transporters (MCTs) to sustain intra-axonal mitochondrial ATP synthesis. While glycolytic lactate production in OLs is largely iron-independent, the maintenance of OL survival and myelination depends strictly on iron-dependent mitochondrial respiration. As OLs succumb to ferroptosis, metabolic support to axons collapses. Intra-axonal ATP failure impairs fast axonal transport, disrupting the transport of APP and its cleavage enzymes, which in turn accelerates localized Aβ generation and causes synaptic failure in highly myelinated projection neurons [16,76,77,78].

#### 4.1.2. Reciprocal Amplification with Amyloid-β and Tau Pathologies

Rather than acting as an isolated pathway, OL ferroptosis engages in a reciprocal amplification loop with canonical AD pathologies. Extracellular Aβ plaque deposition creates a ROS-rich microenvironment that downregulates FPN1, enhancing intracellular Fe^2+^ retention and predisposing OLs to lipid peroxidation [21,26,42]. Conversely, hyperphosphorylated Tau disrupts microtubule-dependent axonal transport, hindering the lysosomal trafficking of ferritin required for autophagic iron degradation (ferritinophagy) and proper iron sequestration. The resulting accumulation of unsequestered cytosolic iron accelerates OL ferroptosis, transforming localized metabolic stress into a self-propagating neurodegenerative cascade across interconnected neural circuits.

#### 4.1.3. Neuroinflammation and the Cyclic Guanosine Monophosphate–Adenosine Monophosphate Synthase–Stimulator of Interferon Genes (cGAS-STING) Axis Inflammatory Loop

Ferroptotic OL rupture releases toxic damage-associated molecular patterns (DAMPs) into the extracellular space, including redox-active iron, oxidized cardiolipin, and fragmented mitochondrial DNA (mtDNA) [79,80]. Fragmented mtDNA is sensed by cytosolic cyclic guanosine monophosphate–adenosine monophosphate synthase in neighboring microglia and astrocytes, driving the activation of the stimulator of interferon genes pathway [81,82,83]. cGAS-STING activation induces a robust type I interferon response and the release of pro inflammatory cytokines, including tumor necrosis factor-α (TNF-α) and Interleukin-1β (IL-1β) [4,84]. These inflammatory mediators downregulate System Xc− and GPX4 expression in surviving adjacent OLs, suppressing their antioxidant defenses and rendering them highly susceptible to secondary ferroptosis [85]. This inflammatory feedback loop propagates white matter degeneration and reinforces the neurodegenerative momentum in AD.

### 4.2. Some Evidence Proving the Existence of the Iron–Mitochondria–Oligodendrocyte Axis

At the molecular level, recent multi-omics studies have identified specific mitochondrial signatures in AD OLs, including the aberrant expression of SAM and SH3 domain-containing protein 1 (SASH1) and the calcium-binding mitochondrial protein EF-hand domain family member D1 (EFHD1), which are associated with mitochondrial dysfunction and cell death [59,61]. Furthermore, dysregulation of mitochondrial iron transporters, such as upregulation of mitoferrin-1/2 (Mfrn1/2) and downregulation of the iron exporter FPN1, has been validated to drive mitochondrial iron loading [86,87,88,89]. In animal models, oligodendrocyte-specific FPN1 conditional knockout mice exhibit iron retention, leading to severe myelin deficits and behavioral abnormalities that recapitulate AD-like phenotypes [90]. Similarly, in five familial Alzheimer’s disease (5xFAD) mice, iron overload precedes plaque formation and is associated with downregulation of myelin-associated genes (including Mbp and Plp1) and the upregulation of ferroptosis markers such as TfR1 and ACSL4 [91,92,93,94].

In clinical studies, high-resolution snRNA-seq of post-mortem AD patient brains reveals a distinct population of “disease-associated oligodendrocytes” characterized by impaired iron handling and mitochondrial stress signatures [60,71]. Neuroimaging using QSM have further demonstrated a strong spatial correlation among regional iron deposition, impaired white matter integrity, and cognitive decline in patients with early-stage AD [95,96,97,98,99].

## 5. Diagnosis and Treatment Frontiers: Multimodal Biomarkers for “Ferroptosis-Positive” Alzheimer’s Disease

### 5.1. Targeted Regulation of Brain Iron Homeostasis Imbalance

Therapeutic strategies targeting iron dysregulation within the IMOA axis have evolved from systemic chelation toward precision modulation of brain iron kinetics (Table 1). Although classical iron chelators such as deferoxamine (DFO) and deferiprone (DFP) have shown efficacy in reducing brain iron load and alleviating oxidative stress in preclinical models [21,100,101], their clinical translation to human AD therapy faces significant barriers, including systemic toxicity, limited blood–brain barrier (BBB) penetration, and detrimental off-target effects on physiological iron handling. Crucially, the recent Deferiprone to Delay Dementia phase 2 clinical trial (NCT03234686) demonstrated that systemic administration of Deferiprone did not slow cognitive decline in AD patients and even accelerated cognitive deterioration in certain measures. This landmark outcome is highly instructive and consistent with the complexities of the IMOA framework; while excess labile iron is neurotoxic, systemic iron chelation indiscriminately deprives OLs of the physiological iron strictly required for mitochondrial respiration, iron–sulfur cluster biogenesis, and ongoing remyelination. This underscores that broad-spectrum chelation is biologically counterproductive [102]. Recent advances have focused on nanotechnology-based delivery systems that encapsulate chelators to enhance CNS bioavailability and reduce peripheral toxicity [29,103], shifting the paradigm from systemic depletion toward precise, targeted iron redistribution. Furthermore, novel therapeutic strategies are being developed to modulate iron transport proteins and restore iron balance. TfR1 and DMT1, to inhibit excessive iron uptake into OLs while simultaneously upregulating the iron exporter FPN1, represent a promising approach to relieve intracellular iron overload [10,90,104]. Additionally, the use of hepcidin mimetics to regulate systemic iron entry into the CNS offers a potential strategy to limit the expansion of the “toxic iron pool” that drives neurotoxicity [24].

### 5.2. Protect Oligodendrocyte Mitochondrial Function and Promote Oligodendrocyte Survival and Myelin Regeneration

To halt AD progression, targeting the OL ferroptosis pathway constitutes a promising therapeutic strategy. Lipophilic small-molecule radical-trapping antioxidants, including Ferrostatin-1 and Liproxstatin-1, directly scavenge peroxyl radicals within lipid bilayers and curb the propagation of lipid peroxidation in OLs. Selective ACSL4 inhibitors suppress the esterification of long-chain PUFAs into membrane phospholipids, depriving ferroptotic cascades of critical lipid substrates [105]. Furthermore, activation of GSH-independent ferroptosis suppressor protein 1-Coenzyme Q10 (CoQ10) axis or upregulation of nuclear factor erythroid 2-related factor 2 (Nrf2)-mediated transcription of GPX4 and FPN1 reinforces endogenous antioxidant defense networks [106,107,108]. Such interventions sustain OL viability, maintain myelin integrity, and alleviate cognitive deficits in AD mouse models. Preserving mitochondrial integrity in OLs is critical for maintaining metabolic support to axons. Emerging therapies employ mitochondrial protective agents such as Mitoquinol mesylate (MitoQ) and Elamipretide, which selectively target the inner mitochondrial membrane to scavenge ROS and inhibit cardiolipin peroxidation [4,20,109]. A breakthrough strategy involves mesenchymal stem cell (MSC)-mediated mitochondrial transfer, where functional mitochondria are delivered from MSCs to injured OLs, restoring bioenergetics and reducing apoptosis [110,111]. Moreover, targeting the mitochondrial iron transporter Mfrn1/2 to prevent mitochondrial iron overload is crucial for ISC biogenesis and respiratory chain function [61,86]. Recent studies also highlight the potential of Nrf2 activators such as dimethyl fumarate to enhance mitochondrial antioxidant defenses and promote mitochondrial biogenesis in glial cells [79,96,112].

To reverse the progression of Alzheimer’s disease, promoting oligodendrocyte survival and remyelination represents a promising therapeutic direction. This suggests that anti-ferroptosis strategies that directly inhibit the ferroptotic cascade in OLs offer a powerful disease-modifying approach for AD. Small molecule ferroptosis inhibitors such as Liproxstatin-1 and Ferrostatin-1 have shown remarkable efficacy in preventing OLs death and preserving myelin integrity in AD models by blocking lipid peroxidation [26,27,43,113]. Novel therapeutic targets include the G protein-coupled receptor 119 (GPR119)-ACSL4 axis. Recent findings reveal that microbiota-derived metabolites such as lysophosphatidylcholine (LPC) can activate GPR119 to inhibit ACSL4, a key enzyme in ferroptosis execution, thereby protecting OLs [34]. Furthermore, promoting the differentiation of OPCs into mature, myelinating OLs is essential for myelin repair. Pharmacological modulation of the wingless/integrated-β-catenin (Wnt/β-catenin) and mitogen-activated protein kinase/extracellular signal-regulated kinase (ERK/MAPK) signaling pathways, as well as treatment with celastrol, has been shown to overcome the differentiation block and enhance remyelination in the iron-overloaded AD brain [32,63,64,114].

**Table 1:** Evidence levels and limitations of selected interventions relevant to the proposed IMOA.

Intervention/Evidence	Population or Model	Reported Outcome	Major Limitation/Translational Meaning
Deferiprone: phase 2 randomized AD trial [102]	81 participants with amyloid-confirmed MCI/early AD; 53 active, 28 placebo; 15 mg/kg orally twice daily for 12 months; MRI completers *n* = 33.	NTB group-by-time β = −0.50 (95% CI −0.80 to −0.20); hippocampal QSM β = −0.68 ppb (95% CI −1.27 to −0.09). Neutropenia: 4/53 (7.5%).	Cognitive harm despite QSM reduction. 54/81 completed treatment. No cell-specific iron measure; imaging subset small. Does not support use for AD.
Deferiprone: non-AD preclinical study [115]	Male C57BL/6J mice; focal lysolecithin-induced optic nerve demyelination; daily intraperitoneal treatment for 3 or 7 days.	Reduced demyelination and glial responses, with axonal/retinal ganglion cell protection reported.	Acute focal injury, not AD; outcomes cannot override the negative human trial. Not proof of oligodendrocyte-specific action.
Ferrostatin-1: AD-related organoid study [26]	PSEN1-ΔE9 patient-derived cortical organoids and isogenic controls; reported benefit at day 50.	Reduced lipid peroxidation and Aβ-related pathology; improved iron-storage measures.	Developmentally immature, mixed-cell preparation; no mature oligodendrocyte-specific efficacy or human clinical endpoint.
Ferroptosis inhibition: non-AD OPC study [85]	Hemin-exposed OPCs and mouse intraventricular hemorrhage.	OPC rescue in vitro and reduced white matter injury with improved neurological outcomes in vivo.	Preclinical Acute heme-associated injury differs from chronic AD. CNS delivery, long-term safety, and lineage specificity require testing.
*B. ovatus*/lysophosphatidylcholine: AD-model study [34]	5xFAD mice; human fecal/serum comparisons were observational.	Reduced mouse Aβ burden and improved cognitive measures; GPR119–ACSL4-related mechanism reported.	Human associations are not treatment evidence. No established oligodendrocyte-targeted clinical mechanism; formulation and CNS exposure remain relevant.
ERK1/2 inhibitor SCH772984: AD-model study [71]	Male AppNL-G-F mice; systemic treatment for 4 weeks; complementary primary-cell experiments.	Improved myelin-related measures and behavioral outcomes; reduced Aβ-related pathology.	Systemic signaling effects do not isolate oligodendrocyte causality; male amyloid model; no clinical AD efficacy demonstrated.
Facilitating cholesterol transport: APOE4-model study [116]	Human tissue and induced pluripotent stem cell-derived cells; intervention in APOE4 mice.	Improved myelination and learning/memory in treated mice.	Supports a lipid-trafficking route, not an iron-specific mechanism. Human efficacy and long-term therapeutic safety remain unestablished.

Evidence categories refer to the disease and model actually studied, not to approval status in unrelated indications. NTB: neuropsychological test battery; QSM: quantitative susceptibility mapping; ppb: parts per billion; MCI: mild cognitive impairment; CI: confidence interval; OPC: oligodendrocyte precursor cell. The NTB interaction estimate is reported on the standardized cognitive z-score scale; lower scores indicate worse performance. Preclinical outcomes are summarized directionally, not as pooled or comparable effect sizes. No numerical preclinical effect size has been inferred from figure appearance. The table is not a ranking of efficacy or a treatment recommendation.

## 6. Future Perspective

The proposed method fundamentally shifts AD paradigms beyond classic amyloid and tau pathologies, positioning white matter metabolic starvation as a vital precursor to grey matter atrophy [5,117]. Degenerative changes in cells may be similar to those in the human body, not just a matter of metabolism, but also possibly due to insufficient energy to support the normal functioning of the cells [118]. Emerging mechanistic evidence—including the regulatory role of long non-coding RNAs like Malat1 under tauopathic stress [11,38]—indicates that oligodendrocyte ferroptosis is not merely a downstream consequence, but a primary driver of Aβ plaque compaction and tau propagation via microglial cross-seeding [4,22]. This newly defined “Iron–Lipid–Energy–Inflammation” cascade is further exacerbated by Aβ-and tau-mediated disruptions in endoplasmic reticulum-to-mitochondria calcium fluxes, locking oligodendrocytes in a bioenergetic crisis that fuels intracellular iron retention [119,120].

Translating these pathophysiological insights into clinical practice requires highly advanced diagnostic frameworks. Integrating snRNA-seq with spatial transcriptomics now allows for the precise mapping of iron-laden oligodendrocyte subpopulations at cellular resolution [31,38]. When coupled with multi-omics fluid biomarker screening [121,122], ultra-high-field quantitative MRI (qMRI) and AI-driven multimodal deep learning frameworks—Alzheimer‘s disease diffusion model (AD-Diff) and Alzheimer‘s disease multi-view multimodal diagnostic network (ADMV-Net) enable the non-invasive tracking of ferroptotic signatures and metabolic profiles, facilitating the precise stratification of prodromal AD patients [34,110,111,113,114].

Despite these promising preclinical and diagnostic advances, therapeutic translation faces formidable hurdles. Successfully disrupting the self-perpetuating IMOA cycle necessitates overcoming the BBB via advanced exosome- or receptor-mediated transcytosis vectors [35,99,123]. Moreover, interventions must achieve exquisite target specificity to effectively chelate pathological CNS iron without disrupting the physiological systemic iron metabolism required for erythropoiesis [79] and critical oligodendrocyte remyelination. Future disease-modifying strategies must adopt combinatorial approaches—integrating targeted iron chelation, mitochondrial protection, and anti-inflammatory agents [65,124,125]—while clearly acknowledging how the IMOA intersects with other prominent non-amyloid frameworks, such as neurovascular unit dysfunction and peripheral-central neuroimmune crosstalk. While earlier concepts involving interventions like cellular reprogramming remain highly speculative and face immense translational barriers, maintaining a focus on restoring glial metabolic homeostasis provides a rigorous and realistic path forward. Ultimately, validating these multi-targeted, IMOA-guided strategies in multi-arm clinical trials remains paramount for establishing the next generation of precision therapeutics for AD.

## 7. Conclusions

The IMOA constitutes a fundamental metabolic triad that maintains white matter integrity; in Alzheimer’s disease, this triad collapses into a vicious cycle of iron overload, mitochondrial failure, and oligodendrocyte ferroptosis. The IMOA has established a new bridge between ferroptosis and AD pathogenesis. As a novel pathological paradigm, the IMOA shifts the focus from protein aggregation to metabolic dysregulation in AD.

Beyond providing target directions for investigating AD pathology, the IMOA also offers new insights into AD therapeutic strategies. Importantly, the IMOA identifies actionable targets for AD, where combinatorial therapeutic approaches—integrating iron chelation with mitochondrial protection—hold promise for halting neurodegenerative progression. Ultimately, IMOA can help us break free from the limitations of the amyloid-β and Tau hypotheses, and it will pave the way for innovative, multi-target diagnostic and therapeutic architectures that address the complex biological reality of AD.

## Data Availability

No new data were created or analyzed in this study. Data sharing is not applicable to this article.

## Abbreviations

5xFAD	five familial Alzheimer’s disease
ACSL4	acyl-CoA synthetase long chain family member 4
AD	Alzheimer’s disease
AD-Diff	Alzheimer’s disease diffusion model
ADMV-Net	Alzheimer’s disease multi-view multimodal diagnostic network
APP	amyloid precursor protein
Aβ	amyloid-β
BBB	blood–brain barrier
cGAS-STING	cyclic guanosine monophosphate–adenosine monophosphate synthase–stimulator of interferon genes
CoQ10	Coenzyme Q10
CSF	cerebrospinal fluid
DAMPs	damage-associated molecular patterns
DAO	disease-associated oligodendrocyte
DFO	deferoxamine
DFP	deferiprone
DMT1	divalent metal transporter 1
EFHD1	EF-hand domain family member D1
ERK/MAPK	mitogen-activated protein kinase/extracellular signal-regulated kinase
FPN1	ferroportin 1
GPR119	G protein-coupled receptor 119
GPX4	glutathione peroxidase 4
GSH	glutathione
IL-1β	Interleukin-1β
IMOA	iron–mitochondria–oligodendrocyte axis
IRE	iron responsive element
IRP	iron regulatory protein
ISC	iron–sulfur cluster
LINGO-1	leucine rich repeat and Ig domain containing 1
LPC	lysophosphatidylcholine
LPCAT3	lysophosphatidylcholine acyltransferase 3
MBP	myelin basic protein
MCTs	monocarboxylate transporters
Mfrn1/2	mitoferrin-1/2
MitoQ	mitoquinol mesylate
MOG	myelin oligodendrocyte glycoprotein
MSC	mesenchymal stem cell
mtDNA	mitochondrial DNA
NFTs	neurofibrillary tangles
Nrf2	nuclear factor erythroid 2-related factor 2
OL	oligodendrocyte
OPC	oligodendrocyte precursor cell
PD	Parkinson’s disease
PET	positron emission computed tomography
PLP	proteolipid protein
p-tau	phosphorylated tau
PUFAs	polyunsaturated fatty acids
qMRI	quantitative magnetic resonance imaging
QSM	quantitative susceptibility mapping
ROS	reactive oxygen species
SASH1	SAM and SH3 domain-containing protein 1
